# Factors influencing left ventricular ejection fraction in patients with coronary microvascular disease and obstructive coronary artery disease

**DOI:** 10.1186/s13104-020-05008-2

**Published:** 2020-03-16

**Authors:** Henry Anselmo Mayala, Magesa Mafuru, Abdalah Mkangala, Mark Mayala, Pedro Pallangyo, Dickson Minja, Mohamed Janabi, Wang Zhao-hui

**Affiliations:** 1 Tongji Medical College, Huazhong University of Science and Technology, 1037 Luoyu Road, Wuhan, 43000 Hubei China; 2 Jakaya Kikwete Cardiac Institute, P. O. Box 65141, West Upanga, Kalenga Street, Ilala District, Dar es Salaam, Tanzania; 3 Muhimbili University of Health and Allied Science, Upanga, Ilala District, Dar es Salaam, Tanzania

**Keywords:** Coronary artery microvascular dysfunction (CMVD), Coronary flow reserve (CFR), Left ventricular ejection fraction (LVEF), Low density lipoprotein- cholesterol (LDL-c), B-type natriuretic peptide (BNP)

## Abstract

**Objective:**

The aim of our research was to evaluate the relationship involving left ventricular ejection fraction, low density lipoprotein, B-type natriuretic peptide, Troponin I and coronary flow reserve, and to determine the predictors of left ventricular ejection fraction in patients with coronary microvascular disease and obstructive coronary artery disease, and in patients with coronary microvascular disease.

**Results:**

The mean age was 58.5 ± 12.5 years. In patients with obstructive coronary disease and coronary microvascular disease we found low density lipoprotein-c had significant inverse relationship with left ventricular ejection fraction, left ventricular ejection fraction also had significant negative relationship with B-type natriuretic peptide, and Troponin-I. While a significant direct relationship turned out to be observed linking left ventricular ejection fraction with coronary flow reserve. Left ventricular ejection fraction had significant negative relationship with low density lipoprotein, and B-type natriuretic peptide in patients with obstructive coronary artery disease only. Age, blood pressure, lipid levels, red cell distribution width, glycated hemoglobin, symptoms, New York heart association classification, alcohol drinking, hypertension, diabetes mellitus, troponin levels and B-type natriuretic peptide were the predictors for left ventricular ejection fraction in coronary microvascular disease patients.

## Introduction

Coronary artery disease is a major non communicable disease problem around the world. The coronary vessels damage can be caused by an array of crucial risk factors such as hypertension, dyslipidemia, diabetes mellitus and smoking cigarettes [1, 2]. Even though there is detailed documentation and guidelines emphasizing administration of secondary prevention medication, an under usage of these medication was observed, meaning that many patients with coronary artery disease did not attain the treatment objective for secondary prevention [3]. Epidemiology studies have revealed men to be more affected by obstructive coronary artery disease compared to women. Moreover, female patients are believed to have more symptom burden and a higher rate of functional disability but a lower prevalence of obstructive coronary artery disease. In patients with coronary artery disease, men tend to have a higher lipid core compared with women [4].

Previous studies were done to investigate serum total cholesterol in relation to left ventricular ejection fraction and coronary artery disease where they found out higher total cholesterol and high density lipoprotein are associated with higher left ventricular ejection fraction [5, 6]. There were no studies done in evaluating the low density lipoprotein, B-type natriuretic peptide, Troponin-I and coronary flow reserve in association with left ventricular ejection fraction in patients with obstructive coronary artery disease and coronary microvascular disease.

In our study, we used a prospective clinical observational design to investigate the association between left ventricular ejection fraction and low density lipoprotein, B-type natriuretic peptide, Troponin-I, and coronary flow reserve and to determine the predictors of left ventricular ejection fraction.

## Main text

### Methodology

#### Study population

We recruited 40 patients attending Union hospital for the first time divided into two sub-group where by half had coronary microvascular disease and the other half had obstructive coronary artery disease.

#### Study design

A prospective clinical observational study.

#### Inclusion criteria

- ST-T dynamic variations on ECG (ST segment desolation, symmetrical T wave reversion, or dynamic change that appears at the time when the chest discomfort occurs).
- Coronary artery examination by coronary angiography was accomplished.
- 18–79 years of age.
- We recruited patients who were showing up for the initial appointment to our medical institution and not in any treatment at all.

#### Exclusion criteria

- Acute myocardial infarction.
- Patients who had percutaneous coronary intervention and those who had coronary artery bypass graft.
- A further cardiac disorders affecting ventricular wall motion or cardiac ejection function, such as stress cardiomyopathy, hypertrophic cardiomyopathy, dilated cardiomyopathy, myocarditis, myocardial amyloidosis.
- Severe arrhythmias such as permanent atrial fibrillation, recurrent and poorly controlled ventricular arrhythmias.
- Severe valvular heart disease.
- Follow up patients on medications including statins.
- Allergic habitus.
- Patients or their family members refused to participate in the study.

#### Definition of terms

- Coronary microvascular dysfunction: ST-segment depression or T-wave inversion on ECG but had TIMI 3 flow on Coronary angiography.
- Obstructive coronary artery disease: ST-segment depression or T-wave inversion on ECG and either TIMI I or II flow on CAG.

#### Study objective

The intention of this research was evaluating the relationship between left ventricular ejection fraction and low density lipoprotein, brain natriuretic peptide, Troponin I and coronary flow reserve, and to determine the predictors of left ventricular ejection fraction in patients with coronary microvascular disease and obstructive coronary artery disease.

#### Image acquisition

PET-CT scan was used to measure coronary flow reserve and assess the microvascular coronary perfusion. The images were obtained using a dedicated PET/CT scanner (Discovery VCT^®^, GE medical systems, Milwaukee WI, USA) immediately after intravenous injection of 3.75 to 5.55 MBq/kg of 13N-NH3, rest and ATP-stressed respectively.

A cutoff CFR value for our study was 2.6 [7].

#### Statistical analysis software

The statistical analysis was done using IBM SPSS Statistics for Windows, Version 20.0. Armonk, NY: IBM Corp, USA.

#### Statistical analysis

Baseline patient characteristics were summarized. All data are presented as mean ± SD for continuous variables and n (%) for categorical variables. Comparisons between groups were made using Pearson correlation or Spearman’s rho for continuous variables and Fisher exact test for categorical variables. A P-value of < 0.05 was considered statistically significant. A multivariate linear regression model was done to determine the predictors of LVEF.

#### Ethical clearance

The clinical protocol and the informed consent forms were approved by the ethics committee of Tongji medical college of Huazhong University of science and technology. All patients read and signed the published informed consent. This clinical study was conducted according to the revised declaration of Helsinki concerning biomedical research in using patient information.

### Results

#### Patients demographic and clinical characteristics

Forty patients participated in our study, whereby 20 patients had coronary microvascular dysfunction and 20 patients had obstructive coronary artery disease. The mean age was 58.5 ± 12.5 years. Approximately 60% of the patients were women. The mean left ventricular ejection fraction was 56.7 ± 7.9, and the mean coronary flow reserve was 2.04 ± 0.56 respectively. The patient’s demographic and clinical characteristics are summarized in Table 1.

**Table 1:** Demographics and clinical characteristics of patients (n = 40)

Characteristics	CMVD (n = 20)	OCAD (n = 20)
Age (years)^a^	48.45 ± 12.7	58.45 ± 12.7
Females, n (%)	12 (60)	12 (60)
Smokers, n (%)	8 (40)	12 (60)
Alcoholic, n (%)	6 (30)	12 (60)
Hypertensive, n (%)	9 (45)	17 (85)
Diabetic, n (%)	3 (15)	13 (65)
Chest pain, n (%)	10 (50)	8 (40)
Chest tightness, n (%)	9 (45)	1 (5)
Difficulty in breathing, n (%)	1 (5)	7 (35)
NYHA (Class I), n (%)	16 (80)	4 (20)
NYHA (Class II), n (%)	4 (20)	8 (40)
NYHA (Class III), n (%)	0 (0)	6 (30)
NYHA (Class IV), n (%)	0 (0)	2 (10)
Systolic blood pressure (mmHg)^a^	131.5 ± 20.0	141.7 ± 21.0
Diastolic blood pressure (mmHg)^a^	81.75 ± 12.8	83.45 ± 20.0
LDL-C (µmol/l)^a^	3.5 ± 1.6	3.5 ± 1.7
HDL-C (µmol/l)^a^	1.18 ± 0.46	1.15 ± 0.35
Troponin-I (pg/ml)^a^	13.1 ± 9.4	24.9 ± 20.0
Red cell distribution width (%)^a^	14.9 ± 3.2	16.0 ± 3.3
Glycated hemoglobin A1C^a^	5.7 ± 1.8	6.9 ± 1.7
Brain natriuretic peptide (pg/ml)^a^	50.3 ± 38.0	176.2 ± 98.9
Ejection fraction (%)^a^	61.5 ± 6.4	51.9 ± 6.4
Coronary flow reserve^a^	1.67 ± 0.28	2.42 ± 0.51

*NYHA* New York Heart Association, *LDL-C* low density lipoprotein cholesterol, *HDL-C* high density lipoprotein cholesterol

^a^Data are expressed as mean ± standard deviation

#### Relationship between left ventricular ejection fraction, low density lipoprotein, brain natriuretic peptide, Troponin-I and coronary flow reserve in patients with coronary microvascular disease and obstructive coronary artery disease

**Fig. 1:**
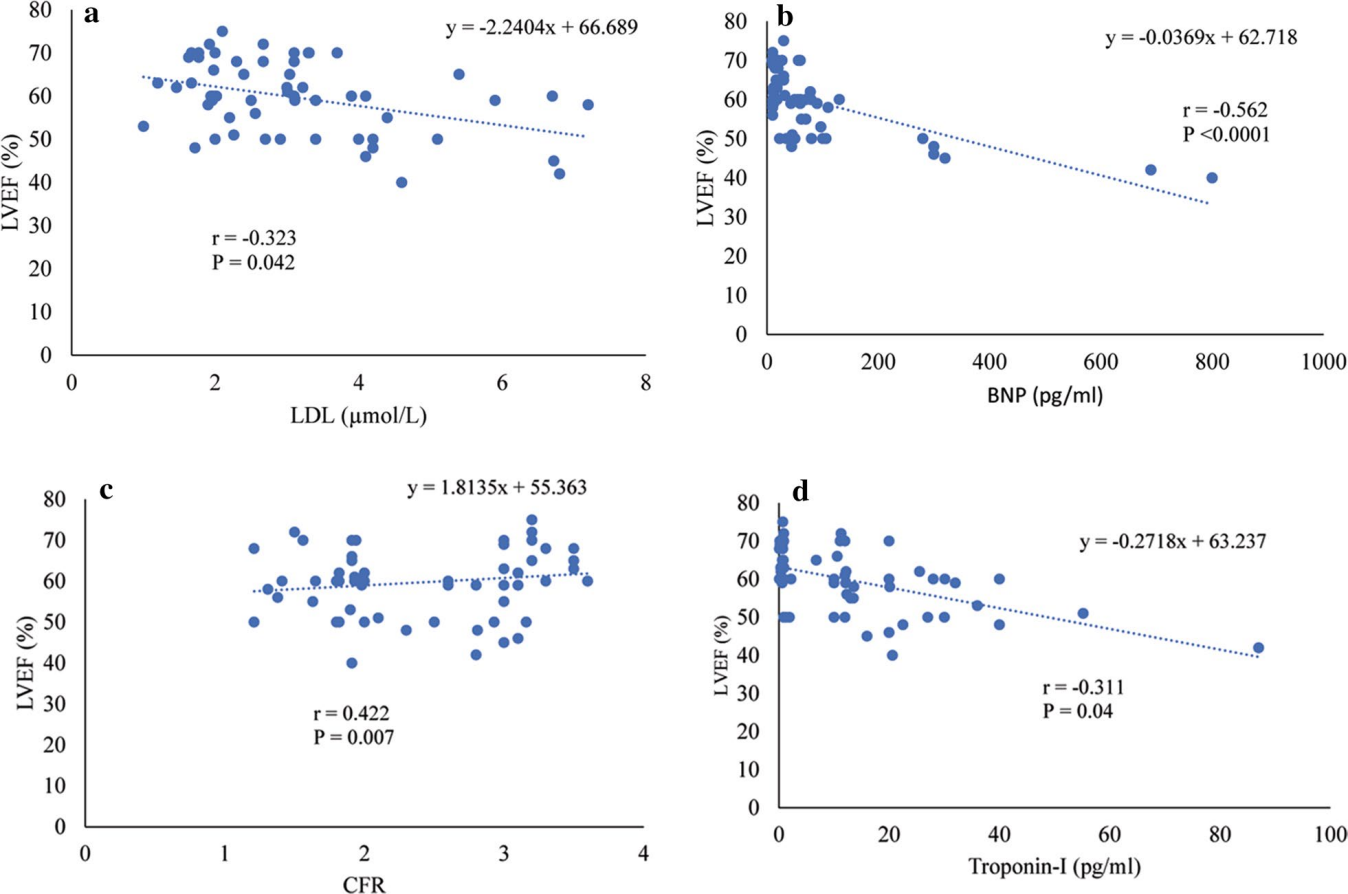
**a** A negative correlation between LVEF (%) and LDL-C (µmol), **b** a negative relationship between LVEF (%) and BNP (pg/ml), **c** a positive correlation between LVEF (%) and CFR, and lastly, **d** a negative relationship between LVEF (%) and Troponin I (pg/ml)

We hypothesized that the factors influencing left ventricular ejection fraction for patients with coronary microvascular disease and obstructive coronary artery disease to be LDL-C, BNP, Troponin-I and CFR. We conducted correlation tests using Spearman’s rho to assess the relationship between LVEF, LDL-C, BNP, Troponin-I and CFR in patients with CMVD and OCAD (Fig. 1a–d). Low density lipoprotein-c (LDL-c) had significant inverse relationship with LVEF (r = − 0.323, P = 0.042), LVEF also had significant negative relationship with BNP (r = − 0.562, P < 0.0001), and Troponin-I (r = − 0.311, P = 0.04). While a significant positive relationship was observed between LVEF and CFR (r = 0.422, P = 0.007).

#### Relationship between left ventricular ejection fraction, low density lipoprotein and brain natriuretic peptide in patients with obstructive coronary artery disease

Left ventricular ejection fraction had significant negative relationship with LDL-C, and BNP. We observed fewer factors influencing the LVEF when we separated OCAD from CMVD patients, and there was no correlating factor in CMVD subgroup.

#### Determination of predictors of left ventricular ejection fraction

i. In patients with coronary microvascular disease and obstructive coronary artery disease

A backward multivariate linear regression model was done for determination of predictors of LVEF in patients with CMVD and OCAD. In this study, the variables Age, symptoms, NYHA classification and BNP qualified to enter the model. After adjusting for confounders, the patients age (coefficient β = 0.19, 95% CI 39.5–58.6, P = 0.023), Difficulty in breathing (coefficient β = − 6.95, 95% CI (− 11.9) to (− 2.0), P = 0.007), NYHA Class III (coefficient β = − 7.14, 95% CI (− 12.55) to (− 1.74), P = 0.011), NYHA Class IV (coefficient β = − 17.25, 95% CI (− 26.18) to (− 8.32), P < 0.0001), and BNP (coefficient β = − 0.03, 95% CI (− 0.042) to (− 0.019), P < 0.0001) were determined as predictors of LVEF in patients with CMVD and OCAD.

ii. In patients with coronary microvascular disease.

After adjusting for confounders, the patients age (coefficient β = 1.31, 95% CI 1.07–1.55, P < 0.0001), systolic blood pressure (coefficient β = − 0.58, 95% CI (− 0.76) to (− 0.41), P < 0.0001), diastolic blood pressure (coefficient β = − 1.71, 95% CI (− 2.14) to (− 1.28), P < 0.0001), HDL (coefficient β = − 4.8, 95% CI (− 8.5) to (1.14), P = 0.02), HbA1c (coefficient β = 2.69, 95% CI 1.66–3.74, P = 0.001), Chest tightness (coefficient β = 33.3, 95% CI 26.4–40.2, P < 0.0001), difficulty in breathing (coefficient β = 12.3, 95% CI 6.93–17.7, P = 0.002), NYHA class I (coefficient β = 20.4, 95% CI 13.3–27.5, P = 0.001), alcohol (coefficient β = 20, 95% CI 15.6–24.6, P < 0.0001), hypertension (coefficient β = 57, 95% CI 47–67, P < 0.0001), Diabetes mellitus (coefficient β = − 64, 95% CI (− 77) to (− 51), P < 0.0001), Troponin I (coefficient β = − 1.65, 95% CI (− 1.9) to (− 1.3), P < 0.0001), and BNP (coefficient β = 0.35, 95% CI 0.24–0.46, P = 0.001) were determined as predictors of LVEF in patients with CMVD (Table 2).

**Table 2:** Multivariate linear regression model investigating the predictors of LVEF in patients with CMVD (n = 20)

Predictors	Multivariate final model		
	Coefficient β ± SE	P-value	95% confidence interval
Constant	109.99 ± 5.47	< 0.0001	95–124
Age	1.31 ± 0.09	< 0.0001	1.07–1.55
Systolic BP (mmHg)	(− 0.58) ± 0.07	< 0.0001	(− 0.76) to (− 0.41)
Diastolic BP (mmHg)	(− 1.71) ± 0.17	< 0.0001	(− 2.14) to (− 1.28)
HDL (µmol/l)	(− 4.8) ± 1.4	0.02	(− 8.5) to (− 1.14)
RDW	2.15 ± 0.21	< 0.0001	1.6 ± 2.7
HbA1c	2.69 ± 0.4	0.001	1.66–3.74
Chest tightness	33.3 ± 2.69	< 0.0001	26.4–40.2
Difficulty in breathing	12.3 ± 2.09	0.002	6.93–17.7
NYHA class I	20.4 ± 2.7	0.001	13.3–27.5
Alcohol	20 ± 1.75	< 0.0001	15.6–24.6
Hypertension	57 ± 3.9	< 0.0001	47–67
Diabetes mellitus	(− 64) ± 5	< 0.0001	(− 77) to (− 51)
Troponin I	(− 1.65) ± 0.13	< 0.0001	(− 1.9) to (− 1.3)
BNP (pg/ml)	0.35 ± 0.043	0.001	0.24–0.46

*LVEF* left ventricular ejection fraction, *CMVD* coronary microvascular dysfunction, *HDL* high density lipoprotein, *RDW* red cell distribution width, *HbA1c* glycated hemoglobin, *BNP* brain natriuretic peptide

### Discussion

The current research indicated a negative correlation between LVEF and LDL-c, BNP and Troponin-I in patients with OCAD and CMVD. To the best our knowledge, this is the first research to reveal the relationship between LVEF and biomarkers.

Previous studies have shown that hyperlipidemia adversely influenced the left ventricular ejection fraction, particularly in patients with myocardial infarction. They went further and detailed an important positive correlation between left ventricular ejection fraction and high density lipoprotein-cholesterol, suggesting that HDL-cholesterol might influence left ventricular systolic function through extra-atherosclerotic mechanisms because they observed left ventricular ejection fraction was adversely influenced by dyslipidemia irrespective of the severity of coronary atherosclerosis [8–11]. There was another study in which, they investigated the association between lipid profile levels and right ventricular volume overload in congestive heart failure, where they revealed lipid levels were inversely correlated to right ventricular end diastolic diameter and right atrium [12]. In our study, which involved patients with obstructive coronary artery disease and coronary microvascular dysfunction, we found out LDL-c to be inversely correlated with left ventricular ejection fraction and it was statistically significant. Meaning one-unit change increase in LDL-c is associated with a unit decrease in LVEF percentage. This shows our study concur with previous study findings.

Several studies have revealed a negative correlation between BNP and LVEF. They revealed that BNP levels were low in patients with heart failure with preserved ejection fraction compared to patients with heart failure with reduced ejection fraction. In another study, they also discovered an important correlation between NT-proBNP and LVEF in elderly patients, whereby worsening LVEF had a significant correlation with NT-proBNP levels [13, 14]. In our study, we also found a negative correlation between left ventricular ejection fraction and B-type natriuretic peptide in patients with obstructive coronary artery disease and coronary microvascular dysfunction (r = − 0.562, P < 0.0001) and in patients with OCAD alone (r = − 0.472, P = 0.035), meaning that one-unit change increase in BNP was related with a unit decrease in LVEF percentage.

Furthermore, another biomarker Troponin-I is a power indicator of myocardial necrosis, it has been studied before and was found to be inversely correlated with left ventricular ejection fraction especially in patients after first myocardial infarction. They found out that the left ventricular ejection fraction of < 50% was predicted by troponin I concentration of > 6.6 ng/ml. In another research analysis, it showed that patients with severe left ventricular systolic dysfunction (LVEF < 35%) had the highest level of troponin I and vice versa. They also showed that the LVEF had a negative correlation with troponin I levels (r = − 0.54, P = 0.001). Despite the above fact, there was an exploration of troponin T, whereby it was also revealed that there was a negative correlation between troponin T levels and LVEF (r = − 0.72, P ≤ 0.0001) [15–17]. In our findings, we showed that there was a significant negative relationship between troponin I and left ventricular ejection fraction in patients with OCAD and CMVD (r = − 0.311, P = 0.04). Meaning one-unit change increase in troponin I levels was associated with a unit decrease in LVEF percentage.

Moreover, there were studies done in the area of determination of predictors of LVEF. Whereby, their analysis demonstrated that several traditional and easily available factors were associated with a greater risk of heart failure development, even among low-risk CAD population. Some of the studies showed lipids predicted the severity of new onset CAD in type-2 DM patients and not in relation to LVEF, differentiating from our study. In another research, low HDL-c was strongly predictive of cardiovascular events in patients with coronary artery disease [18–20]. In our research we also found out that traditional risk factors influenced the LVEF concurring the previous studies even though we were the only one, who further evaluated patients with OCAD and CMVD. In the current published articles indicated the relevance of inflammatory biomarkers particularly CRP and Pentraxin 3 as the prognostic indicators of coronary artery disease congruent with our findings even though we assessed different biomarkers [21, 22].

We believe by sharing these findings of our study, will empower the clinicians with knowledge on coronary microvascular dysfunction in relation to OCAD, by exploring the relationship between LVEF and biomarkers, and predictors of LVEF in these patients.

## Conclusion

There was a strong negative relationship between left ventricular ejection and biomarkers, with a significant positive association between LVEF and CFR.

## Limitation

A small sample size was our study limitation.

## Data Availability

Data and materials are available upon request to the authors.

## Abbreviations

LVEF: Left ventricular ejection fraction
LDL-C: Low density lipoprotein-cholesterol
BNP: B-type natriuretic peptide
CFR: Coronary flow reserve
CMVD: Coronary microvascular disease
OCAD: Obstructive coronary artery disease
